# The Role of Leptin and Adiponectin in Obesity-Associated Cognitive Decline and Alzheimer’s Disease

**DOI:** 10.3389/fnins.2018.01027

**Published:** 2019-01-14

**Authors:** Leticia Forny-Germano, Fernanda G. De Felice, Marcelo Nunes do Nascimento Vieira

**Affiliations:** ^1^Institute of Medical Biochemistry Leopoldo de Meis, Federal University of Rio de Janeiro, Rio de Janeiro, Brazil; ^2^Centre for Neuroscience Studies, Department of Psychiatry, Queen’s University, Kingston, ON, Canada

**Keywords:** adipokine, leptin, adiponectin, obesity, Alzheimer’s disease, cognitive decline

## Abstract

Cross-talk between adipose tissue and central nervous system (CNS) underlies the increased risk of obese people to develop brain diseases such as cognitive and mood disorders. Detailed mechanisms for how peripheral changes caused by adipose tissue accumulation in obesity impact the CNS to cause brain dysfunction are poorly understood. Adipokines are a large group of substances secreted by the white adipose tissue to regulate a wide range of homeostatic processes including, but not limited to, energy metabolism and immunity. Obesity is characterized by a generalized change in the levels of circulating adipokines due to abnormal accumulation and dysfunction of adipose tissue. Altered adipokine levels underlie complications of obesity as well as the increased risk for the development of obesity-related comorbidities such as type 2 diabetes, cardiovascular and neurodegenerative diseases. Here, we review the literature for the role of adipokines as key mediators of the communication between periphery and CNS in health and disease. We will focus on the actions of leptin and adiponectin, two of the most abundant and well studied adipokines, in the brain, with particular emphasis on how altered signaling of these adipokines in obesity may lead to cognitive dysfunction and augmented risk for Alzheimer’s disease. A better understanding of adipokine biology in brain disorders may prove of major relevance to diagnostic, prevention and therapy.

## Introduction

Lifestyle, nutrition and lack or inefficient exercise to control body weight is making the global population more susceptible to obesity. Food with high sugar and carbohydrate contents are in general cheaper and more readily available for the current busy lives. These factors contribute to a widespread epidemic of overweight and obesity, metabolic disorders characterized by the accumulation of peripheral and/or visceral adipose tissue (Hendrickx et al., 2005; Hill et al., 2012). Obesity is a well established risk factor for a number of other chronic disorders, including type 2 diabetes, cardiovascular diseases, arthritis and some types of cancer (Elias et al., 2005; Haslam and James, 2005; Lengyel et al., 2018). More recently, attention has been drawn to the impact of obesity on central nervous system (CNS) functioning, as growing evidence indicate that the obese population are more susceptible to some neurological conditions. Overweight and, in particular, central obesity during midlife have been associated to a higher risk of developing cognitive disorders, including Alzheimer’s disease (AD), later in life (Kivipelto et al., 2005; Rosengren et al., 2005; Whitmer et al., 2005, 2008). In addition, overweight and obese individuals are at higher risk for developing mood disorders, such as major depression disorder (MDD) and bipolar disorder (BD) (Luppino et al., 2010; reviewed in Mather et al., 2009; Vogelzangs et al., 2010; Vannucchi et al., 2014; Mansur et al., 2015). These evidences indicate that changes in the organism that accompany overweight and obesity can ultimately lead to CNS dysfunction. However, the pathophysiological mechanisms and molecular players underlying this connection are poorly known.

The obese body accumulates adipose tissues broadly classified in white (WAT) and brown adipose tissue (BAT) (Caron et al., 2018). Initially considered a fat storage organ, it is now established that the WAT is an endocrine organ secreting a group of substances that act in a pleiotropic manner exerting autocrine, paracrine or endocrine effects on processes in the periphery and CNS (Ahima, 2012). This class of WAT-derived substances are named adipocytokines or adipokines. Adipokines comprehend a wide range of molecules including hormones, cytokines and growth factors (Lehr et al., 2012), and exert a variety of distinct functions in the organism ranging from control of metabolism homeostasis to immune system regulation and behavior (Kiliaan et al., 2014; Fasshauer and Blüher, 2015). Abnormal production and secretion of adipokines resulting from aberrant accumulation of WAT in obesity leads to dysregulation of important homeostatic systems, resulting in the complications of obesity and in an increased risk for comorbidities such as insulin resistance and type-2 diabetes, hypertension, atherosclerosis and other cardiovascular diseases, and neurological disorders such as depression and AD (Kiliaan et al., 2014).

In the following sections, we review the literature for studies on the role of adipokines as the possible mediators of signals from the periphery to the CNS in obesity-associated brain dysfunctions. Specifically, we will focus on two of the most abundant and well studied adipokines – leptin and adiponectin – and their interaction with cognitive processes of the CNS in health and disease.

## Adipokines in Obesity and Related Diseases

Disproportional accumulation of white adipose tissue in overweight and obesity is accompanied by a generalized change in the circulating levels of several adipokines. Adipose dysfunction and adipokine dysregulation are thought to be responsible for or, at least, be an important contributor to the increased risk of obese people to develop a number of related diseases. For instance, increased levels of proinflammatory adipokines such as interleukin (IL)-1β, IL-6, TNFα and leptin, and decreased levels of anti-inflammatory adipokines, such as adiponectin, in obesity produce a chronic state of low-grade inflammation which promotes the development of insulin resistance and type-2 diabetes, hypertension, atherosclerosis and other cardiovascular diseases, and some types of cancer (Friedemann et al., 2012; Odegaard and Chawla, 2013; Hotamisligil, 2017). Moreover, since adiponectin also acts as an insulin-sensitizing hormone in muscle and liver, lower levels of adiponectin further contribute to peripheral insulin resistance in obesity (Liu et al., 2016; Saltiel and Olefsky, 2017). Lastly, increased circulating levels of leptin in obesity lead to hypothalamic leptin resistance, turning down anorexigenic and energy expenditure signals and further contributing to aggravate obesity (Waterson and Horvath, 2015).

The CNS is not exempt from negative effects of obesity, as adipose dysfunction associated with obesity have been linked to altered brain metabolism, neuroinflammation, neuronal dysfunction, brain atrophy, impaired mood and cognitive decline (Luppino et al., 2010; Ahima et al., 2017; Arshad et al., 2018). Studies associating obesity to morphometric changes in brain structure are somehow controversial. While the vast majority demonstrates obesity to be associated with lower gray matter and whole brain volumes (Pannacciulli et al., 2006; Gunstad et al., 2007; Raji et al., 2010; Yokum et al., 2011; Marqués-Iturria et al., 2013; Veit et al., 2014), fewer publications showed no association (van Boxtel et al., 2007; Sharkey et al., 2015). This controversy has been recently addressed in a meta-analysis study which found obesity to be consistently associated with lower gray matter volumes in brain areas associated with executive functions, including medial prefrontal cortex, left temporal lobe and bilateral cerebellum. These findings were further validated in the same study in an independent dataset (García-García et al., 2018).

Obesity has also been linked to cognitive disorders. Obese individuals are under greater risk to develop age-related cognitive decline, vascular dementia, mild cognitive impairment (MCI) and AD (Frisardi et al., 2010). Furthermore, animal models of obesity also develop cognitive decline (Winocur et al., 2005; Kleinert et al., 2018; McLean et al., 2018). Mechanisms proposed to underlie obesity-associated risk for cognitive disorders include development of brain inflammation (Nguyen et al., 2014; Heneka et al., 2015) and central insulin resistance (de la Monte et al., 2009; Kim and Feldman, 2015). Peripheral inflammation in obesity results from secretion of proinflammatory cytokines by adipocytes and adipose tissue-resident activated macrophages (Lee and Lee, 2014). Proinflammatory cytokines such as TNFα, interleukin (IL)-1β and IL-6, have been shown to cross the blood–brain barrier (BBB) (Banks, 2005) and may act in concert with proinflammatory factors produced locally by microglial cells to foster brain inflammation in AD (De Felice and Ferreira, 2014). Importantly, such cytokines has been shown to modulate synaptic plasticity and cognition both in health and disease states (Nelson et al., 2012; Gruol, 2015; Rizzo et al., 2018). Moreover, as occurs in peripheral tissues in obesity and type 2 diabetes, proinflammatory cytokines, in particular TNFα, mediate the development of neuronal insulin resistance (Bomfim et al., 2012; Lourenco et al., 2013). Since both insulin and cytokine signaling in the brain regulate synaptic plasticity, learning and memory, neuroinflammation and neuronal insulin resistance may be key mediators of obesity-associated cognitive decline (reviewed in De Felice and Ferreira, 2014).

The increased risk for obese individuals to develop CNS pathology reflects the capacity of adipose tissue to communicate with the brain and impact brain function. It is not clear yet how this cross-talk occurs, but growing evidence indicate that adipokines are involved. Adipokines may impact brain physiology through different mechanisms. Some adipokines such as leptin and TNFα can cross the BBB and act directly in the brain while other adipokines would act on endothelial brain cells, regulating BBB permeability and the access of other circulating mediators into the brain. Importantly, in pathological states such as inflammation, the BBB integrity is compromised allowing the penetration of adipokines and other substances to which the brain is normally inaccessible. Finally, local expression of adipokines such as leptin and adiponectin have been reported in the mammalian brain (Denver et al., 2011; Thundyil et al., 2012). Advances on adipokine research have been providing information to understand how obesity affect brain function to cause brain atrophy, cognitive dysfunction, mood disorders and increase the risk for neurological diseases.

**FIGURE 1:**
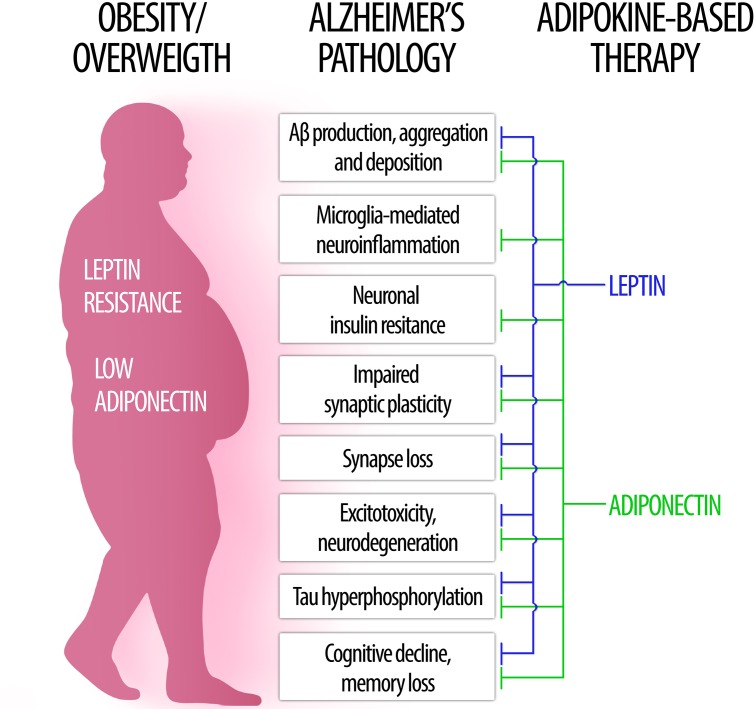
The role of leptin and adiponectin in Alzheimer’s disease physiopathology. (Left) In obesity, low levels of circulating adiponectin as well as central leptin resistance may contribute to brain pathology and increased risk for Alzheimer’s disease. (Right) Conversely, therapeutic approaches based on leptin (blue) and adiponectin (green) signaling may counteract a wide range of pathological processes associated to AD (center).

In the following sections, we will examine key aspects of leptin and adiponectin – the two most abundant and well studied adipokines – regarding their roles in brain physiology and their involvement in obesity-related cognitive dysfunction, dementia and AD (Figure 1).

## Leptin

### Leptin in the Brain

The discovery of leptin in 1994 is considered the cornerstone for adipokine research (Zhang et al., 1994). Leptin is classically related to the central control of food intake and energy homeostasis (Friedman, 2016). However, other neurophysiological functions have been attributed to leptin, including brain development (Ahima et al., 1998; Bouret, 2010) neurogenesis (Garza et al., 2008), neuronal protection (Doherty et al., 2013), mood and stress regulation (Lu et al., 2006; Paz-Filho et al., 2010), reproduction and reproductive behaviors (Caprio et al., 2001; Lu et al., 2006; Chehab, 2014).

Leptin is mainly produced by adipose tissue, more specifically by visceral white adipocytes in rodent and by subcutaneous adipose tissue in human (Trayhurn et al., 1995; Hube et al., 1996). However, local expression of leptin mRNA and protein in CNS has been reported (Morash et al., 1999; Scott et al., 2009; Patterson et al., 2011). Circulating levels of leptin are dynamic and susceptible to different regulatory factors such as metabolism, body fat mass, circadian cycle and sexual dimorphisms (Trayhurn et al., 1995; Licinio et al., 1997; Saad et al., 1997; Park and Ahima, 2015). Leptin crosses the BBB by binding to specific receptors or interacting with BBB. The partially saturate influx of leptin through BBB indicates that membrane proteins facilitate leptin uptake where the entrance is limited by transport dynamics (Banks et al., 1996). There is evidence that leptin transport into the brain can be mediated by megalin at the choroid plexus epithelium (Dietrich et al., 2008; Bartolome et al., 2017) and by short LepR isoforms in tanycytes (Hileman et al., 2002; Balland et al., 2014; Di Spiezio et al., 2018). Therefore, levels of leptin in the brain are subject to regulation by local leptin production, circulating leptin levels and leptin transport across BBB through different mechanisms.

Leptin receptors (LepR or ObR) were first detected in 1995 at the mouse choroid plexus (Tartaglia et al., 1995). Posterior studies revealed that alternative splicing during LepR-coding *db* gene expression result in six different LepR isoforms (LepRa-f) (Lee et al., 1996; Wang et al., 1996). Long leptin receptor (LepRb) is the isoform which best bind to leptin and activate intracellular pathways (Allison and Myers, 2014). LepRs are tyrosine-kinase receptors that undergo autophosphorylation upon leptin binding to activate different signaling cascades, including JAK/STAT, ERK/MAPK and IRS/PI3K pathways. STAT (signal transducer and activator of transcription) is a family of transcription factor which, upon phosphorylation, migrate to the nucleus and regulate transcription of target genes such as the suppressor of cytokine signaling 3 (SOCS3) and protein tyrosine phosphatase 1B (PTP1B). STAT3 is the major signaling pathway recruited by leptin and, due to its responsive property, STAT3 phosphorylation became an alternative way to identify leptin-responsive cells and infer leptin sensitivity (Ramos-Lobo and Donato, 2017; Pan and Myers, 2018).

LepRb is the main isoform of leptin receptor expressed in the brain. Robust expression comprehends the arcuate nucleus (ARC), the paraventricular nucleus and the dorsomedial, lateral and ventromedial regions of the hypothalamus (Schwartz et al., 1996; Wauman and Tavernier, 2011; Mercer et al., 2018). Extrahypothalamic sites also express leptin receptors including hippocampus, cortex, midbrain and hindbrain (Elmquist et al., 2005; Patterson et al., 2011). A transgenic mouse expressing LepRb-cre in combination with cre-inducible enhanced green fluorescent protein (EGFP) and farnesylated EGFP (EGFPf) was used to reveal a detailed distribution of LepRb expressing neurons as well as their projection sites throughout the brain (Patterson et al., 2011). Regions found to contain LepRb expressing neurons were consistent with those previously determined by examination of LepRb mRNA expression via *in situ* hybridization (Elmquist et al., 1998), but this study further identified projections sites of LepRb neurons in the brain. LepRb neurons and projection were observed throughout the prefrontal cortex, while more discrete, circumscribed regions containing both LepRb neurons and projections were found in insular, temporal, auditory, somatosensory and visual cortices (Patterson et al., 2011). The hippocampus displayed few LepRb neurons along with projections from LepRb neurons mostly concentrated in CA1–3 regions and, to a lower extent, in the dentate (Elmquist et al., 2005; Mercer et al., 2018).

The wide variety of leptin effects in the CNS likely reflects the peculiar distribution of leptin receptors through different brain structures as well as the particular signaling cascades activated by these receptors in distinct regions (Bjørbaek and Kahn, 2004).

In the following sections, we review key aspects of brain leptin signaling that are relevant for its role in cognitive decline and AD (Table 1).

**Table 1:** Summary of the main findings associating leptin signaling to physiopathological processes relevant to AD.

Finding	Model(s)	Reference
Leptin signaling modulates synaptic function and plasticity	Hippocampal slices from adult rats	Shanley et al., 2001; Xu et al., 2008; Moult et al., 2009, 2010; Luo et al., 2015; McGregor and Harvey, 2017; McGregor et al., 2018
LepR-deficiency causes impairment of LTP and spatial memory	Zucker rats and db/db mice	Li et al., 2002
Leptin treatment improves learning and memory	Hippocampal slices from adult rats; rats ICV-infused with Aβ; AD mouse models (SAMP-8 mice; CRND8 transgenic mice (TgCRND8) and APP/PS1	Shanley et al., 2001; Farr et al., 2006; Oomura et al., 2006; Greco et al., 2010; Pérez-González et al., 2014; Tong et al., 2015
Deficient leptin transport across BBB	Aged mice, APP/PS1 mice	Carro et al., 2005; Dietrich et al., 2008
PTP1B upregulation	APP/PS1 mice; Human AD patients	Pei et al., 1994; Mody et al., 2011; King et al., 2018
SOCS3 upregulation	APP/PS1 mice; human AD patients	Walker et al., 2015; Iwahara et al., 2017; King et al., 2018
Impaired leptin signaling	Human AD patients; Tg 2576 mice; APP/PS1 mice	Bonda et al., 2014; Maioli et al., 2015
Decreased leptin levels in CSF and plasma	Human AD patients	Holden et al., 2009; Lieb et al., 2009; Bigalke et al., 2011; Chakrabarti, 2014; Johnston et al., 2014; Khemka et al., 2014b; Baranowska-Bik et al., 2015
Increased leptin levels in CSF and brain	Human AD patients	Bonda et al., 2014; Yin et al., 2018
Unchanged leptin levels in CSF and brain	Human AD patients	Maioli et al., 2015; Oania and McEvoy, 2015
Aβ disrupts leptin signaling	Human AD patients; rabbit hippocampal slices	Marwarha et al., 2010; Bonda et al., 2014
Leptin reduce Aβ levels	SH-SY5Y cells; Neuro2a cells; primary neuronal cultures from rat embryos; APP/PS1 mice; adult rats	Fewlass et al., 2004; Pérez-González et al., 2014; Tong et al., 2015
Leptin reduce tau hyperphosphorylation	SH-SY5Y cells; NT2 cells; primary rat cortical neurons	Greco et al., 2008


### Leptin in Synaptic Function and Memory

Synaptic plasticity is a crucial event for learning and memory process. Long-term potentiation (LTP) and long-term depression (LTD) of synaptic transmission are pivotal mechanisms in hippocampal memory formation and consolidation (Bliss and Collingridge, 1993). At the molecular level, LTP and LTD are regulated by insertion or removal of NMDA- and AMPA-type glutamate receptors from post-synaptic terminals, thereby modulating synaptic transmission strength (Malenka and Bear, 2004).

There is substantial evidence indicating that leptin regulates hippocampal synaptic plasticity and memory. LepRs in hippocampal neurons are closely associated with somato-dendritic and synaptic regions, indicating the potential for leptin to modulate synaptic function (Harvey, 2015). Recent studies demonstrate that leptin activation of JAK2/STAT3 signaling pathway induces transcription of genes related to LTD (McGregor et al., 2017). Moreover, learning and memory formation are modulated by JAK2/STAT5 signaling (Furigo et al., 2018), a pathway also subject to regulation by leptin receptor signaling in the hippocampus (Gong et al., 2007). Two different LepR-deficient rodent strains (Zucker rats and db/db mice) show impaired LTP in the CA1 region of the hippocampus, which is accompanied by poor performance in the water maze spatial memory test (Li et al., 2002). Conversely, leptin treatment has been shown to improve learning and memory performances in different models (Shanley et al., 2001; Oomura et al., 2006; reviewed in Irving and Harvey, 2013).

Leptin was shown to regulated synaptic plasticity by targeting NMDA- and AMPA-receptors trafficking, particularly in the CA1 region of the hippocampus. Schaffer collaterals (SCs) and temporoammonic (TA) projections to CA1 pyramidal neurons exhibit an age-dependent response to leptin. Interestingly, these functionally distinct circuits appear to respond differently to leptin exposure. In SC-CA1 synapses, leptin was shown to induce depression of synaptic transmission in young rodents hippocampus (Xu et al., 2008; Moult et al., 2009) whereas, in adult animals, leptin promote synaptic strengthening and LTP (Moult et al., 2010; Moult and Harvey, 2011). Different effects were observed in TA-CA1 synapses, where leptin induces LTP in young (Luo et al., 2015), promote LTD in the adult but had no effect on synaptic transmission in aged rat hippocampus (McGregor et al., 2018). Importantly, the age- and regional-variability of leptin effects on hippocampal synaptic plasticity appears to be driven by the composition of NMDARs subtypes, and to be achieved by regulating the traffic of different subsets of AMPARs toward and away from synapses. Leptin regulation of synaptic plasticity involves several downstream targets, including JAK2-STAT3, PTEN, PI3K, ERK, and CaMKII (for more comprehensive reviews, see Moult and Harvey, 2009; McGregor and Harvey, 2017).

### Leptin Resistance

Several studies demonstrated that leptin responsiveness decreases with obesity, aging and neurodegenerative diseases, a phenomenon called leptin resistance. Leptin resistance affects a range of processes such as food intake, insulin sensitivity, inflammation and cognition. In obesity, leptin resistance leads to increased production of leptin by adipocytes and hyperleptinemia, in an attempt of the organism to compensate for low leptin responsiveness. Decreased leptin signaling in the CNS may be related to defective leptin transport across BBB, LepR downregulation and/or deficient leptin signaling downstream LepRs (Myers et al., 2008, 2012; de Git and Adan, 2015; Bluher, 2016; Banks et al., 2018).

Triglycerides can impair BBB leptin transport causing central leptin deficiency (Banks et al., 2004). Furthermore, it was recently demonstrated that triglycerides can cross the BBB to directly induce hypothalamic leptin and insulin receptor resistance, leading to decreased satiety and cognitive impairment in mice (Banks et al., 2018). Interestingly, triglycerides increased leptin binding in different brain regions, suggesting an allosteric or post-receptor rather than a competitive mechanism of inhibition of LepR signaling by triglycerides (Banks et al., 2018). In light of longitudinal studies linking increased mid-life triglyceride levels to the risk for AD (Vemuri et al., 2017; Nägga et al., 2018), the above results suggest that triglycerides may contribute to AD pathogenesis and progression by suppressing leptin signaling in the brain. Deficient leptin transport across BBB by megalin leading to reduced leptin entry into the brain has also been described in aged mice and in mouse models of AD (Carro et al., 2005; Dietrich et al., 2008).

At the intracellular level, leptin signaling is negatively regulated by the suppressor of SOCS3 and by the PTP1B. SOCS3 binds to LepR and JAK2 to inhibit their activities, whereas PTP1B dephosphorylates tyrosine residues deactivating LepR and JAK2. PTP1B have been linked to central leptin resistance in humans (Myers et al., 2010) as well as in a variety of animal models of obesity (Cheng et al., 2002; Zabolotny et al., 2002; White et al., 2009) and aging (Morrison et al., 2007). SOCS3 and PTP1B were also found upregulated in the brains of AD mouse models (Mody et al., 2011; Iwahara et al., 2017; King et al., 2018) and AD patients (Pei et al., 1994; Walker et al., 2015). Therefore, targeting PTP1B and SOCS3 may prove valuable to overcome central leptin resistance in obesity, aging, and AD (Engin, 2017; Vieira et al., 2017).

Central leptin resistance can also be mediated by downregulation of LepRs expression. In this regard, LepR levels were found to be decreased in the hippocampus of AD patients, whereas leptin levels were upregulated both in CSF and locally in the hippocampus, possibly due to a compensatory mechanism for receptor dysfunction (Bonda et al., 2014; Maioli et al., 2015). Age-dependent changes in LepR expression levels were also reported in Tg2576 and APP/PS1 mouse models of AD (Maioli et al., 2015; King et al., 2018). Although the results from these studies are hard to interpret, they are consistent with an impact of AD phenotypes in LepR expression in brain areas relevant to cognition and memory.

Taken together, the results described above indicate that impaired brain leptin signaling may play a role in AD pathophysiology, and that restoring leptin signaling may constitute a valid approach to restore synaptic function and cognition in AD.

### Leptin Signaling, Obesity, and Alzheimer’s Disease

Several studies indicate that over-weight and obesity during mid-age increases risk to develop AD (Whitmer et al., 2008; Hassing et al., 2009; Xu et al., 2011; Gustafson et al., 2012; Emmerzaal et al., 2014; Alhurani et al., 2016). In contrast, high weight in late-life was shown to be protective against AD and cognitive decline (Hughes and Ganguli, 2009; Gustafson et al., 2012; Emmerzaal et al., 2014; Bell et al., 2017). Surprisingly, weight loss in late age is related to higher risk for dementia and AD (Barrett-Connor et al., 1996; Buchman et al., 2005; Stewart et al., 2005; Johnson et al., 2006; Hughes and Ganguli, 2009; Gao et al., 2011; Joo et al., 2018). This apparently paradoxical influence of body weight on dementia and AD risk is far from being understood. Interestingly, a similarly complex relation is observed between overweight/obesity and other neurodegenerative diseases. For instance, in amyotrophic lateral sclerosis (ALS) patients, mild obesity is associated with increased survival rates, whereas morbid obesity increases mortality rates (Paganoni et al., 2011; Gallo et al., 2013). The influence of body weight on the clinical outcomes of dementia, AD and other neurodegenerative diseases suggest that adipose tissue dysfunction and adipokines dysregulation may play a broad role across the spectrum of neurodegenerative diseases. Moreover, the explanation for this puzzling and somehow paradoxical influence of adiposity on brain diseases may lie in how weight changes impact production and action of adipokines such as leptin and adiponectin.

Evidence regarding how leptin levels are affected in AD are controversial. Several studies show decreased leptin levels in CSF and plasma in AD patients (Holden et al., 2009; Lieb et al., 2009; Bigalke et al., 2011; Chakrabarti, 2014; Johnston et al., 2014; Khemka et al., 2014b; Baranowska-Bik et al., 2015) whereas increased leptin levels (Bonda et al., 2014; Yin et al., 2018) and unaffected levels (Maioli et al., 2015; Oania and McEvoy, 2015) in CSF and cerebral tissue were also reported. Conflicting findings can be associate to variations in dementia score, post-mortem neuropathological analyses to confirm dementia, sample size, stratification in sex, age, and others (Kiliaan et al., 2014; Mcguire and Ishii, 2016).

Leptin signaling was found to interact with several mechanisms associated with AD physiopathology. Aβ disrupt leptin signaling leading to down-regulation of hippocampal leptin and LepR expression (Marwarha et al., 2010; Bonda et al., 2014). Conversely, leptin was reported to be neuroprotective in AD models by suppressing Aβ accumulation and toxicity and attenuating tau pathology. Leptin administration reduce Aβ levels in the Tg2576 mouse model of AD (Fewlass et al., 2004). The anti-amyloidogenic effect of leptin involve inhibition of APP processing by down regulating β-amyloid precursor protein (APP) cleaving enzyme (BACE1) and increase in APOE-dependent Aβ uptake, and seems to be mediated by activation of AMP-activated protein kinase (AMPK) (Fewlass et al., 2004; Marwarha et al., 2010; Pérez-González et al., 2014; Tong et al., 2015).

Tau phosphorylation can be suppressed by leptin modulation of GSK3β activity (Greco et al., 2008). Importantly, leptin treatment also improve memory performance in different mouse models of AD (Farr et al., 2006; Greco et al., 2010; Pérez-González et al., 2014). In a rat model of intracerebroventricular Aβ injection, chronic leptin administration rescued Aβ-induced impairment of spatial memory and late-phase LTP (Tong et al., 2015). Leptin was further shown to enhance hippocampal neurogenesis in AD mice (Pérez-González et al., 2011). Collectively, the above studies provide initial validation for the potential therapeutic applications of leptin signaling enhancement in AD brains.

## Adiponectin

Adiponectin is a 30 KDa adipokine encoded by the AdipoQ gene, mainly produced and secreted by adipocytes and highly abundant in human plasma. Adiponectin is known to increase insulin sensitivity of target organs such as liver and muscle, ultimately regulating peripheral glucose and fatty acid metabolism (Hotta et al., 2001; Yamauchi et al., 2001, 2002). Besides being a metabolic regulator, adiponectin is also known for its anti-inflammatory and anti-oxidant activity (Takemura et al., 2007; Liu Y. et al., 2015). These characteristics make adiponectin a protective factor in conditions such as obesity, type 2 diabetes and cardiovascular diseases (Yamauchi et al., 2003a; Spranger et al., 2006; Antoniades et al., 2009). Levels of circulating adiponectin are decreased in obesity and metabolic syndrome, likely contributing to the development of insulin resistance (Arita et al., 1999; Hotta et al., 2001; Yang et al., 2001). Low levels of adiponectin have also been linked to several types of cancer (Miyoshi et al., 2003; Bao et al., 2013; Ma et al., 2016) and cardiovascular diseases (Komatsu et al., 2004).

Adiponectin naturally self-associate to form different types of aggregates. In plasma, adiponectin exists as trimers, hexamers or high molecular weight (HMW) multimers. Adiponectin trimers are mainly stabilized by non-covalent interactions, whereas larger aggregates require crosslinking between subunits by disulfide bonds (Waki et al., 2003). In addition, adiponectin also circulates as biologically active globular fragment generated through proteolysis of full-length adiponectin (Fruebis et al., 2001). Importantly, these post-translational modifications affect biological activity, as distinct adiponectin complexes present tissue-specificity and may activate different signaling pathways (for reviews, see Wang et al., 2008; Liu and Liu, 2014).

Adiponectin acts through binding to three different receptors: adiponectin receptor 1 (AdipoR1), adiponectin receptor 2 (AdipoR2) and T-cadherin. AdipoR1 and AdipoR2 are the most abundant sites for adiponectin binding and mediate most of adiponectin actions through the organism (Yamauchi et al., 2003b, 2007). Activation of AdipoRs by adiponectin leads to the recruitment of the adaptor protein APPL1 (adaptor protein, phosphotyrosine interacting with PH domain and leucine zipper 1) (Mao et al., 2006) which, in turn, mediates downstream adiponectin signaling through a number of different pathways including AMPK, PI3K-Akt, MAPK-Erk1/2, PPARα, p38-MAPK, PTEN and JNK (Cheng et al., 2007; Chandrasekar et al., 2008; Coope et al., 2008; Lee et al., 2008). APPL1 also plays a crucial role in the insulin sensitization effect of adiponectin by interacting with and facilitating the binding of IRS1/2 to the insulin receptor (Ryu et al., 2014).

### Adiponectin in the Brain

Data from epidemiological studies demonstrate that diabetes, obesity, and metabolic syndrome increase the risk of developing cognitive problems and dementia (Stewart and Liolitsa, 1999; Baker et al., 2011; Kerwin et al., 2011; McCrimmon et al., 2012; Lehtisalo et al., 2016; Espeland et al., 2017; Pal et al., 2018; Palta et al., 2018). More recently, evidence indicate that insulin signaling dysfunction and chronic neuroinflammation are key factors in cognitive decline, MCI and AD (Steen et al., 2005; Arnold et al., 2018, reviewed in De Felice, 2013; Ferreira et al., 2014; Heneka et al., 2015). In face of these observations, adiponectin has gained attention in the context of such CNS disorders due to its potentially protective actions as an anti-inflammatory and insulin sensitizing hormone.

Adiponectin is present in human and mouse CSF, albeit in much lower concentrations than in blood (Kubota et al., 2007; Kusminski et al., 2007; Neumeier et al., 2007). However, the source(s) for adiponectin in CNS are not completely clear. Initial studies suggested that adiponectin expression was limited to adipocytes and, to a lower extent, other peripheral tissues such as liver, muscle, placenta, and epithelium. More recently, however, some studies reported adiponectin expression at both mRNA and protein levels in the chicken and mammalian brain (Maddineni et al., 2005; Hoyda et al., 2007; Kaminski et al., 2014; Shen et al., 2014). Whether peripheral adiponectin crosses the BBB to reach the brain is a matter of debate. While some studies conclude that adiponectin does not cross the BBB (Pan et al., 2006; Spranger et al., 2006), other studies found evidence suggesting that peripheral adiponectin does reach the brain through the BBB (Qi et al., 2004; Yau et al., 2014). This is an important open question with physiological and potential translational relevance, since adiponectin-based therapies to treat brain conditions would possibly consist of peripheral administration and require adiponectin to reach the CNS. Therefore, further studies are required to clarify whether or not peripheral adiponectin can reach the brain tissue through BBB.

Adiponectin receptors are also expressed in different structures of the brain. The hypothalamus is the best documented site for AdipoRs expression in the mammalian brain, but there is also evidence for expression in endothelial brain cells, cerebral cortex, brain stem and hippocampus (Yamauchi et al., 2003b, 2007) reviewed in (Thundyil et al., 2012). Interestingly, a study in post mortem human brains also revealed intense immunostaining for AdipoR1 in the nucleus basalis magnocellularis (NBM) (Psilopanagioti et al., 2009), a small structure rich in cholinergic neurons projecting diffusely to the whole neocortex and other brain areas, and that is severely affected in AD (Liu A.K. et al., 2015).

Adiponectin functions in the brain are highly diversified. It has been shown to act locally in the brain to control key processes of brain physiology including neuronal excitability and synaptic plasticity, neuroprotection, neurogenesis and regulation of glial cells activation (Yau et al., 2014; Chabry et al., 2015; Song et al., 2015; Nicolas et al., 2017; Shah et al., 2017; Pousti et al., 2018). More recently, adiponectin was found to modulate glucose metabolism in hippocampal neurons, increasing glucose uptake, glycolysis and ATP production rates (Cisternas et al., 2018). Adiponectin also acts on brain to regulate peripheral and systemic processes such as thermogenesis, energy expenditure (Qi et al., 2004) and reproduction (Angelidis et al., 2012). Finally, central adiponectin has been shown to regulate behaviors like food intake (Suyama et al., 2016), locomotor activity (Miyatake et al., 2015) as well as cognition, anxiety and mood (Jeong et al., 2012; Zhang et al., 2017; Cao et al., 2018; Cezaretto et al., 2018; Nicolas et al., 2018; Platzer et al., 2018; Sun et al., 2018). This variety of central effects of adiponectin likely reflects the wide distribution of different adiponectin receptors throughout the brain. For the purpose of this review, the following sections will focus on roles of adiponectin that are most likely relevant to cognitive dysfunction and AD, namely synaptic regulation, insulin sensitivity, neuroinflammation, neuroprotection and neurogenesis (Table 2).

**Table 2:** Summary of the main findings associating adiponectin signaling to physiopathological processes relevant to AD.

Finding	Model(s)	Reference
Adiponectin and AdipoRs signaling modulates synaptic function and plasticity	APP/PS1 mice; NSE-APPsw mice; adiponectin-deficient mice; rats	Zhang et al., 2016, 2017; Shah et al., 2017; Pousti et al., 2018; Yoon et al., 2018
Adiponectin deficiency causes AD-like synapse loss and memory impairment	Adiponectin-deficient mice	Ng et al., 2016
Adiponectin signaling improves memory	Adiponectin-deficient mice; APP/PS1 mice; NSE-APPsw mice; Aβ i.c.v.-infused mice	Ali et al., 2015; Shah et al., 2017; Zhang et al., 2017
High adiponectin levels is associated with better cognitive performance	Middle-aged non-diabetic humans	Cezaretto et al., 2018
Adiponectin signaling deficiency leads to impaired brain insulin signaling	Adiponectin knockout mice; AdipoR1 deficient mice	Ng et al., 2016; Kim et al., 2017
Adiponectin improve neuronal insulin sensitivity	Insulin resistant SH-SY5Y human neuroblastoma cells	Ng et al., 2016
Adiponectin deficiency promote neuroinflammation	Adiponectin knockout mice	Ng et al., 2016
Adiponectin signaling attenuates microglia-mediated neuroinflammation	Environment-enriched mice; adiponectin deficient mice; mouse model of intracerebral hemorrhage; brain sorted microglia; primary microglial cells; BV2 microglial cells exposed to Aβ	Chabry et al., 2015; Nicolas et al., 2015, 2017; Song et al., 2017; Zhao et al., 2018
Adiponectin and AdipoRs agonists are neuroprotective	Primary rat hippocampal neurons; SH-SY5Y cells; rodent models of hemorrhagic and ischemic stroke	Jung et al., 2006; Chen et al., 2009; Jeon et al., 2009; Qiu et al., 2011; Chan et al., 2012; Song et al., 2013, 2015; Guo et al., 2015; Wang et al., 2016; Yang et al., 2017; Ma et al., 2018
Adiponectin and AdipoRs agonists promotes neurogenesis	Exercised mice; adiponectin deficient mice; corticosterone-induced anxiety/depressive-like mice	Yau et al., 2014; Chan et al., 2017; Nicolas et al., 2018
Adiponectin and AdipoRs agonists has antidepressive properties	Exercised mice; corticosterone-induced anxiety/depressive-like mice	Yau et al., 2014; Zhang et al., 2016; Chan et al., 2017; Nicolas et al., 2018
Adiponectin signaling deficiency produce AD-like phenotypes	Adiponectin knockout mice; AdipoR1 deficient mice	Ng et al., 2016; Kim et al., 2017
Adiponectin signaling impairment in AD models	APP/PS1 mice	Várhelyi et al., 2017
Adiponectin reduce Aβ production and aggregation	APP/PS1 mice; SH-SY5Y cells overexpressing APP	Shah et al., 2017
Adiponectin attenuates tau hyperphosphorylation	Streptozotocin injected rats	Xu et al., 2018
Altered levels of adiponectin in AD (conflicting results have been reported, see the Section “Adiponectin in Alzheimer’s Disease” for details)	Human subjects	Kamogawa et al., 2010; Une et al., 2010; van Himbergen et al., 2012; Teixeira et al., 2013; Khemka et al., 2014a; García-Casares et al., 2016; Ma et al., 2016


### Adiponectin in Synaptic Function and Memory

Synapse loss is the best pathological correlate for clinical manifestations of cognitive dysfunction in AD (DeKosky and Scheff, 1990; Terry et al., 1991). Compelling evidence indicate that Aβ oligomers, toxins that accumulate in AD brains, target synapses impairing synaptic function and plasticity and causing synapse loss (reviewed in Lambert et al., 1998; Ferreira and Klein, 2011; Forny-Germano et al., 2014) Aβ oligomers inhibit LTP and promote LTD of hippocampal synapses *in vitro* and *in vivo* (Wang et al., 2002; Shankar et al., 2008; Li et al., 2009; Jürgensen et al., 2011).

Interestingly, recent data indicate that adiponectin signaling directly regulates synaptic function and plasticity, preserving and enhancing cognitive functions in a number of different models. Intracerebroventricular administration of adiponectin in anesthetized rats potentiates high frequency stimulation (HFS)-induced LTP and suppresses low-frequency stimulation (LFS)-induced LTD. Furthermore, adiponectin administration alone induced a chemical LTP, independent of presynaptic stimulus (Pousti et al., 2018). Osmotin, a plant-derived homolog of adiponectin capable of activate AdipoRs, improves LTP impairment and ameliorates memory deficits in a mouse model of AD (Shah et al., 2017). This effect appears to be mediated by AdipoR1 and the Nogo66 receptor 1 (NgR1) and involve promotion of neurite outgrowth and increasing of dendritic spine and synapse density in the hippocampus (Zhang et al., 2016; Yoon et al., 2018). Adiponectin-deficient mice displays increased excitability of hippocampal dentate gyrus (DG) granule neurons, associated with impaired extinction of contextual fear memory. Adiponectin and its mimetic drug AdipoRon restored fear memory extinction via AdipoR2 activation and inhibition of DG neuron excitability (Zhang et al., 2017). Aged adiponectin-deficient mice have reduced levels of synaptic proteins suggesting synapse loss, and also performs poorly in spatial memory and contextual fear conditioning tests (Ng et al., 2016). Importantly, both adiponectin and osmotin treatments ameliorate learning and memory deficits in AD animal models (Ali et al., 2015; Shah et al., 2017). Caloric restriction increases circulating adiponectin levels and improve cognition in mice probably via regulation of the AMPK signaling pathway in mouse hippocampus (Ma et al., 2018). Finally, a recent clinical study found that individuals with higher adiponectin levels tend to perform better in a delayed word recall test, supporting the notion that adiponectin is a protective factor against cognitive decline and represents a promising therapeutic strategy in cognitive disorders (Cezaretto et al., 2018).

Collectively, these recent studies consistently established a previously ignored ability of adiponectin to regulate hippocampal synaptic function and plasticity and to improve cognitive function, learning and memory. This further support a protective role for adiponectin in AD, suggesting that diminished brain adiponectin signaling in obesity may favor AD onset and progression and that adiponectin signaling may be an interesting target in AD therapy.

### Adiponectin in Central Insulin Signaling

Neuronal insulin signaling is important for synaptic plasticity and memory, mainly by regulating glutamate receptors trafficking (Beattie et al., 2000; Man et al., 2000; Skeberdis et al., 2001; Zhao et al., 2004). Impaired insulin signaling is well documented both in human AD patients and in a variety of AD animal models (Steen et al., 2005; Bomfim et al., 2012; Talbot et al., 2012) and is considered an important mechanism for neuronal dysfunction and cognitive impairment in AD (Ferreira et al., 2014; Vieira et al., 2018) Synaptotoxicity of Aβ oligomers is accompanied by insulin receptor dysfunction *in vitro* and *in vivo* and can be prevented by treatment with insulin sensitizing drugs and by insulin itself (De Felice et al., 2009; Bomfim et al., 2012; Batista et al., 2018). These discoveries encouraged several groups to evaluate the efficacy of different classes of anti-diabetic drugs in AD models, and positive preclinical results paved the way for human clinical trials (for comprehensive reviews, see De Felice, 2013; Yarchoan and Arnold, 2014; de la Monte, 2017). In this context, interest has recently been directed to the insulin-sensitizing actions of adiponectin to correct aberrant insulin signaling in AD. Impaired brain insulin signaling was observed, along with several other AD-like pathological features, in adiponectin knockout mice and in AdipoR1 deficient mice (Ng et al., 2016; Kim et al., 2017). Conversely, adiponectin increases insulin sensitivity in SH-SY5Y neuronal cell line modeling insulin resistance, through AdipoR1 receptor activation of AMPK (Ng et al., 2016). These data indicate that adiponectin has the potential to restore neuronal insulin signaling, with possible therapeutic implications for AD and other neurodegenerative diseases. However, further translational studies using proper animal models of AD are required to validate adiponectin signaling as a therapeutic approach to overcome brain insulin resistance in AD.

### Adiponectin in Neuroinflammation

Adiponectin is well known for its anti-inflammatory activity in peripheral tissues, which include suppression of macrophage activation and secretion of pro-inflammatory cytokines (Yokota et al., 2000; Elfeky et al., 2016, 2018). For that, adiponectin is considered a protective factor against pathological processes such as peripheral insulin resistance and cardiovascular diseases, whereas low levels of adiponectin in obesity contributes to chronic inflammation and obesity-associated risk for related diseases (For recent reviews, see Ohashi et al., 2015; Liu et al., 2016).

Alzheimer’s disease is also characterized by a chronic state of low-grade inflammation in the brain. This response is mediated by microglial cells activation and secretion of pro-inflammatory cytokines TNFα, IL-6, and IL-1β (Bamberger et al., 2003; Lourenco et al., 2013; Heneka et al., 2015; reviewed in De Felice and Lourenco, 2015; Sarlus and Heneka, 2017). Proinflammatory cytokines trigger a series of detrimental events in the AD brain, including neuronal insulin resistance, endoplasmic reticulum stress, synaptotoxicity and neurodegeneration (Bomfim et al., 2012; Lourenco et al., 2013; Rizzo et al., 2018).

Adiponectin-knockout mice develop a series of pathological features in the brain resembling AD, including insulin resistance, reduced levels of synaptic proteins and the presence of neuroinflammatory markers such as microgliosis, astrogliosis and elevated levels of the pro-inflammatory cytokines TNFα and IL-1β (Ng et al., 2016). Environmental enrichment, housing conditions that promote physical activity, cognitive engagement and social interactions, has been shown to improve cognitive functions and be protective in AD mouse models. These effects are at least in part due to modulation of microglial response to the insult of Aβ oligomers (Xu et al., 2016; Vieira and Beckman, 2017). Interestingly, it was recently shown that beneficial effects of environmental enrichment to the brain are mediated by adiponectin, and involves the promotion of an anti-inflammatory activation state of microglia with decreased production of pro-inflammatory cytokines (Chabry et al., 2015; Nicolas et al., 2015). The same group also showed that globular adiponectin directly inhibits microglia pro-inflammatory profile *in vivo* and *in vitro* (Nicolas et al., 2017) in a mechanism involving AdipoR1 and NF-κB. Adiponectin also modulates microglial activation profile under Aβ toxicity *in vitro*, via PPARγ activation (Song et al., 2017). Further evidence for the anti-inflammatory actions of adiponectin in the CNS come from a study showing that CTRP9, an AdipoR1 agonist, attenuates neuroinflammation in a mouse model of intracerebral hemorrhage through a AdipoR1/AMPK/NFκB signaling mechanism (Zhao et al., 2018). Moreover, AdipoRon treatment suppresses macrophage recruitment in a model of spinal cord injury (Zhou et al., 2018). Finally, it has been proposed that adiponectin can also modulate neuroinflammation by reducing expression of pro-inflammatory cytokines by brain endothelial cells (Spranger et al., 2006). These evidences support a role for adiponectin in mitigating brain inflammation, and suggest that adiponectin deficiency in obesity may trigger neuroinflammatory events leading to AD and other related CNS disorders.

### Adiponectin in Neuroprotection and Neurogenesis

Neurodegeneration in AD is mediated by overactivation of glutamate receptors and excessive neuronal calcium influx, a process called excitotoxicity (Dong et al., 2009; Wang and Reddy, 2017; Arshad et al., 2018). Excitotoxicity can be triggered by toxic amyloid-β aggregates *in vitro* and *in vivo*, and represents an important pathological mechanism in synaptic failure and neuronal death in AD (reviewed in Paula-Lima et al., 2013). Excitotoxicity is commonly associated with mitochondrial dysfunction and oxidative stress (Johnston et al., 2014; Bhat et al., 2015), and also occurs in other pathological conditions such as stroke and brain and spinal cord injuries (Li and Stys, 2000; Ahuja et al., 2017).

In the past few years, a growing number of studies demonstrated the neuroprotective properties of adiponectin and other AdipoRs agonists against a variety of neuronal toxic insults *in vitro* and *in vivo*. *In vitro*, adiponectin was shown to protect SH-SY5Y human neuroblastoma cells against oxidative stress and cytotoxicity induced by Aβ and MPP+, an inhibitor of mitochondrial complex I (Jung et al., 2006; Chan et al., 2012). Adiponectin neuroprotection was also observed in a model of kainic acid (KA)-induced excitotoxicity in primary cultures of rat hippocampal neurons (Jeon et al., 2009; Qiu et al., 2011). A series of studies also reported neuroprotective roles of adiponectin and AdipoRs in different models of hemorrhagic and ischemic stroke. Cellular mechanisms reported to underlie adiponectin and AdipoRs neuroprotection include suppression of oxidative stress, apoptosis and inflammation, and involve a remarkable variety of intracellular targets, including antioxidant enzymes, AMPK, JNK/PI3K/Akt, PKA, GSK3β, NFkappaB, Bax/Bcl-2 and caspase 3 (Chen et al., 2009; Song et al., 2013, 2015; Guo et al., 2015; Wang et al., 2016; Yang et al., 2017; Ma et al., 2018). These neuroprotective actions of adiponectin may be therapeutically applicable in neurodegenerative diseases.

The antidepressant effects of physical exercise are well known and widely used in the clinic as a non-pharmacological treatment for depression. Recently, adiponectin was shown to play a crucial role in antidepressant effects of exercise, by mediating exercise-induced hippocampal neurogenesis (Yau et al., 2014; Chan et al., 2017). The neurogenic and neurotrophic effects of adiponectin signaling were further demonstrated by intracerebroventricular injection of adiponectin in adiponectin-deficient mice (Zhang et al., 2016). Remarkably, chronic intraperitoneal administration of the AdipoRs agonist AdipoRon in an anxiety/depression mouse model reversed depression-like state by modulating several CNS processes, including neurogenesis (Nicolas et al., 2018). This study is of particular translational relevance since AdipoRon, a small-molecule adiponectin-mimetic drug, showed central activity while administered peripherally. It has been suggested that, for its neurogenic and antidepressant actions, adiponectin can be explored as a pharmacological surrogate for physical exercise to treat depression and, possibly, other brain disorders (Li et al., 2015).

### Adiponectin in Alzheimer’s Disease

Recent studies indicate that adiponectin signaling deficiency is sufficient to induce an AD-like phenotype in mice. Aged adiponectin-knockout mice recapitulate several aspects of AD pathology, including increased Aβ levels and deposition, tau hyperphosporylation, neuroinflammation, synapse loss, neuronal apoptosis and impaired insulin signaling. Importantly, aged adiponectin knockout mice also performed poorly in spatial memory and fear conditioning behavioral tests (Ng et al., 2016). These observations were further corroborated by another recent study showing that gene-therapy induced suppression of AdipoR1 also produces an AD-like phenotype, which includes impaired spatial memory and learning, increased levels of Aβ aggregates and hyperphosphorylated tau, insulin signaling dysfunction, neuroinflammation and neurodegeneration markers (Kim et al., 2017). Collectively, these studies make strong case for a role for adiponectin deficiency in AD pathogenesis. The NBM is a cholinergic nucleus in the basal forebrain which is severely affected in AD and other neurodegenerative diseases. It is well documented that neuronal loss of NBM cholinergic neurons contributes to cholinergic dysfunction and, most importantly, it correlates with clinical measures of dementia (Arendt et al., 1983; Whitehouse et al., 1986; Iraizoz et al., 1999; reviewed in Liu A.K. et al., 2015). Interestingly, NBM was found to be a prominent site of expression of AdipoR1 (Psilopanagioti et al., 2009). Therefore, it is possible that adiponectin deficiency contributes to the onset and progression of AD by promoting NBM dysfunction and degeneration. This hypothesis, however, remain to be tested.

Conversely, impairment in adiponectin function is also observed in amyloid-based AD models, whereas activating adiponectin signaling reduces AD-like pathology. In APP/PS1 mice, changes in AdipoRs expression levels were less responsive to a stress-inducing paradigm as compared to wild-type mice (Várhelyi et al., 2017). In the same model, the adiponectin-homolog osmotin ameliorated AD-like neuropathological features such as Aβ production and aggregation, synaptic dysfunction and impaired LTP, memory and cognitive deficits. AdipoR1 silencing abolished osmotin beneficial effects and further aggravated brain pathology in AD-mice (Shah et al., 2017). Osmotin was further shown to reduce Aβ deposition in cultured SH-SY5Y human neuroblastoma cells overexpressing APP. Osmotin effects were mediated by activation of AMPK, an enzyme downregulated by Aβ oligomers in hippocampal neurons (Seixas da Silva et al., 2017). In a rat model of streptozotocin-induced brain pathology, intracerebroventricular injection of adiponectin rescued cognitive deficits and attenuated GSK3β-mediated tau hyperphosphorylation in AD-relevant sites (Xu et al., 2018). These results suggest boosting adiponectin signaling, particularly through AdipoR1, as a potential therapeutic approach in AD (Ng and Chan, 2017). In this regard, chronic treatment with donepezil, an acetylcholinesterase inhibitor widely use to treat AD, was recently shown to increase serum levels of adiponectin (Pákáski et al., 2013). Moreover, thiazolidinediones (TZDs) such as rosiglitazone and pioglitazone, PPARγ agonists used for decades to treat type 2 diabetes due to its insulin sensitization activity (Saltiel and Olefsky, 1996; Malinowski and Bolesta, 2000; Hong et al., 2018) were recently repurposed to treat AD. The insulin-sensitizing effect of TZDs is in part mediated by induction of peripheral adiponectin and AdipoRs expression (Yu et al., 2002; Tsuchida et al., 2005; Nie and Li, 2017). Therefore, it is possible that adiponectin mediate part of the observed beneficial effects of donepezil and TZDs.

AdipoRon, an orally bioavailable small-molecule agonist of adiponectin receptors (Okada-Iwabu et al., 2013), was shown to modulate hippocampal synaptic transmission and to facilitate fear memory extinction in rodents (Zhang et al., 2017). AdipoRon was further shown to regulate activity of dopaminergic neurons in the ventral-tegmental area (Sun et al., 2018) and to act as an antidepressant and metabolic regulator in a mouse model of depression (Nicolas et al., 2018). Importantly, central AdipoRon effects were obtained by peripheral administration, and it was shown that it crosses the BBB to activate AdipoRs in the brain. However, to our knowledge, there are no available data on AdipoRon effects in AD models. It should be interesting to investigate the efficacy of this adiponectin-mimetic drug with translational potential in AD models.

Studies relating adiponectin levels to AD in humans are controversial. Increased baseline adiponectin levels in plasma have been associated with a higher risk of women, but not men, to develop AD and other types of dementia (van Himbergen et al., 2012). Furthermore, elevated adiponectin levels were reported in the plasma and CSF of subjects with MCI and sporadic AD (Une et al., 2010; Khemka et al., 2014a), whereas plasma levels of adiponectin positively correlated with the degree of dementia. The correlation between high blood adiponectin and AD has been replicated and supported by meta-analysis study (Ma et al., 2016). However, while the studies described above suggest increased adiponectin levels to be associated with AD and dementia, opposite results have also been reported. One study found lower levels of circulating adiponectin in MCI and AD subjects. Moreover, adiponectin levels failed to predict progression of cognitive dysfunction from normal to MCI and from MCI to AD (Teixeira et al., 2013). One larger study also reported low levels of plasma adiponectin to be associated with MCI, even though this association was observed in men, but not in women (Kamogawa et al., 2010). In line with these observations, it has been shown that, in diabetic patients, low plasma levels of adiponectin correlate with lower gray-matter volume and reduced glucose utilization in temporal regions of the brain, similarly to what is observed in AD (García-Casares et al., 2016). Finally, a recent study reports that levels of adiponectin are higher in blood but lower in CSF of AD and MCI patients.

Available data regarding a possible association between adiponectin levels in blood and CSF to MCI and AD are conflicting and inconclusive. Therefore, further studies are warranted to reveal the potential use of adiponectin measurement for diagnostic purposes and its clinical relevance for the physiopathology of these neurological conditions. Worth mentioning, some studies found that the association between adiponectin levels and dementia can be sex-specific (Kamogawa et al., 2010). This may be of particular relevance in the case of AD, where a considerable sexual dichotomy is observed with women being at a significantly higher risk to develop the disease.

## Concluding Remarks

Obesity is pandemic in present days. Beyond its intrinsic complications, and the obvious social and psychological impact, obesity harms extend to a wide range of associated health conditions to which it represents a major risk factor. More recently, growing attention is being given to the impact of obesity on CNS function, as accumulating evidence indicate higher incidence of neurological disorders in the obese population. The mechanisms by which fat accumulation and adipose tissue dysfunction in obesity result in CNS pathology are poorly understood. It is widely accepted that dissection of such mechanisms will greatly improve our knowledge on the cross-talk between peripheral metabolism and brain physiology, and may provide novel targets for therapeutic intervention for prevention and/or treatment of neurological dysfunctions associated with obesity.

Adipokines are secreted factors which carry regulatory signals from adipose tissue through systemic circulation to control a wide range of physiological functions throughout the human body. Not surprisingly, adipokine dysregulation in obesity results in the disruption of homeostasis in a variety of organs and systems, and underlie obesity complications and risk for associated chronic conditions. In this context, adipokines emerged as strong candidates to represent the mediators of pathological signals from adipose tissue to CNS in metabolic disorders.

The studies reviewed here provide evidence supporting a role of leptin and adiponectin, two highly abundant and well characterized adipokines, as key mediators of obesity-related CNS dysfunctions (Figure 1). We found consistent evidence that leptin and adiponectin, as well as their receptors, are present in the brain and function as important regulators of different aspects of brain physiology. Importantly, leptin (Table 1) and adiponectin (Table 2) signaling have been shown to interfere with a range of neuropathological events covering those most commonly present in neurodegenerative diseases and, in particular, in AD. These include amyloidogenesis, tau hyperphosphorylation, neuroinflammation, oxidative stress, endoplasmic reticulum stress, insulin resistance, synaptic dysfunction and cognitive impairment. Remarkably, the phenotype of adiponectin- or adiponectin receptor-deficient mice recapitulates the majority of AD neuropathological hallmarks. Therefore, dysregulated adiponectin and leptin signaling may mediate the detrimental impact of obesity on CNS and raise the risk for cognitive decline and AD. Importantly, restoring proper leptin and adiponectin signaling in the brain may constitute beneficial, disease-modifying therapeutic interventions in such neurological conditions (Figure 1).

## Author Contributions

LF-G and MV wrote the manuscript. MV designed the figure. LF-G, FDF, and MV planned the scope and reviewed the final manuscript.

## Conflict of Interest Statement

The authors declare that the research was conducted in the absence of any commercial or financial relationships that could be construed as a potential conflict of interest.
